# 固相支撑液液萃取-液相色谱-串联质谱测定尿液中10种双酚类化合物和5种对羟基苯甲酸酯

**DOI:** 10.3724/SP.J.1123.2024.01001

**Published:** 2024-09-08

**Authors:** Yufan XUE, Ting SHANG, Juntao CUI, Lingjuan ZHAO, Pei LI, Xiangying ZENG, Zhiqiang YU

**Affiliations:** 1.中国科学院广州地球化学研究所,有机地球化学国家重点实验室,广东省环境资源利用与保护重点实验室,广东广州 510640; 1. State Key Laboratory of Organic Geochemistry, Guangdong Provincial Key Laboratory of Environmental Protection and Resources Utilization, Guangzhou Institute of Geochemistry, Chinese Academy of Sciences, Guangzhou 510640, China; 2.中国科学院大学,北京 100049; 2. University of Chinese Academy of Sciences, Beijing 100049, China

**Keywords:** 固相支撑液液萃取, 液相色谱-串联质谱, 双酚类化合物, 对羟基苯甲酸酯, 尿液, solid supported liquid-liquid extraction (SLE), liquid chromatography-tandem mass spectrometry (LC-MS/MS), bisphenols (BPs), parabens (PBs), urine

## Abstract

尿液中双酚类化合物(BPs)和对羟基苯甲酸酯类化合物(PBs)的浓度水平监测为考察其在人体内的暴露提供基础数据,是准确评估其健康风险的前提。本研究使用基于固相支撑液液萃取(SLE)原理的新型萃取柱,建立了新的BPs和PBs快速前处理技术,在此基础上利用液相色谱-串联质谱法(LC-MS/MS)同时测定人体尿液中10种BPs和5种PBs。尿样先酶解,然后经SLE柱富集,使用15 mL乙酸乙酯-正己烷(3∶7, v/v)混合溶液进行洗脱;通过引进水、甲醇和乙腈的三元流动相梯度洗脱系统,实现了15种目标化合物的准确定性和定量分析。在混合尿液基质中,低、中、高3个水平的加标回收率为84.3%~119.8%;除双酚S外,其余14种化合物的基质效应均在20%以下,表明具有良好的回收率和较低的生物基质干扰。15种目标化合物在各自的线性范围内线性关系良好,相关系数均大于0.995;方法定量限为0.03~0.30 μg/L;精密度测试结果显示,日内和日间连续进样仪器响应的相对标准偏差分别为1.4%~8.4%和5.7%~14.6%,证明具有良好的稳定性和重复性。该方法成功应用于10个普通人群尿样中10种BPs和5种PBs的测定。结果表明,检出率最高的化合物为MeP、EtP、PrP和BPA,其中值质量浓度分别为1.10、0.60、0.21和0.55 μg/L,其余化合物检出率低于50%,这可能与化合物的生产使用量、生物可利用性以及在人体内的生物代谢能力相关。

## Abstract (en)

Bisphenols (BPs) and parabens (PBs) are of great concern for environmental pollution and human health because of their endocrine-disrupting effects and potential health hazards. Urinary biomonitoring of BPs and PBs can provide basic data for human internal exposure evaluation, which is a prerequisite for accurately assessing their health risks. In this study, we developed a new pretreatment procedure based on solid supported liquid-liquid extraction (SLE) for the simultaneous separation of ten BPs and five PBs in human urine, followed by high performance liquid chromatography-tandem mass spectrometry (HPLC-MS/MS) analysis. In the instrumental analysis, the HPLC conditions and MS/MS parameters were comprehensively optimized. Accurate qualitative and quantitative determination of ten BPs and five PBs was achieved by introducing a ternary gradient elution system of water, methanol, and acetonitrile for LC separation. During sample pretreatment, the extraction solvent and elution volume were optimized. Specifically, urine samples were held at room temperature and centrifuged at 3000 r/min for 10 min. The supernatant (2 mL) was then transferred to a glass tube, and the pH was adjusted to 5.0 using HCl (0.5 mL; 0.1 mol/L) and NaAc-HAc buffer (1.5 mL). Thereafter, *β*-glucuronidase-arylsulfatase (20 μL) and surrogate standard solutions (10 ng;^13^C_12_-BPS,^13^C_12_-BPAF,^13^C_6_-MeP, and ^13^C_6_-BuP) were added, and the mixture was incubated in a shaker bath in the dark at 37 ℃ for 16 h. After incubation, the hydrolyzed sample (4 mL) was loaded onto an SLE cartridge and equilibrated for a minimum of 5 min to ensure the solution was completely absorbed by the packing material. Subsequently, the target chemicals were eluted with a mixed ethyl acetate/*n*-hexane solution (3∶7, v/v; 15 mL). Separation of the targets was performed on a ZORBAX SB-C18 reversed-phase column (250 mm×4.6 mm, 5 μm) using an acetonitrile-methanol-water system as the mobile phase. The method was verified by spiking mixed urine samples at three levels (1, 5, and 50 μg/L), with the recoveries ranging from 84.3% to 119.8%. Except for bisphenols (BPS), whose matrix effect was calculated as -21.8%, the matrix effects of other analytes were lower than 20%, indicating low matrix interference. The linear ranges of the analytes varied from 0.1-500 μg/L to 1-500 μg/L, with correlation coefficients higher than 0.995. The method limits of quantification for target chemicals ranged from 0.03 to 0.30 μg/L, and the relative standard deviations of intra- and inter-day experiments were 1.4%-8.4% and 5.7%-14.6%, respectively, suggesting high stability and reproducibility. The method was successfully applied to the determination of ten BPs and five PBs in 10 urine samples from a general population. The concentrations of target chemicals in the human urine samples varied. Methylparaben (MeP), ethylparaben (EtP), propylparaben (PrP), and bisphenol A (BPA) were detected in all samples, with median mass concentrations of 1.10, 0.60, 0.21, and 0.55 μg/L, respectively. The detection rates of the other chemicals were less than 50%, which may be related to the production and use of specific chemicals, their bioavailability, and biological metabolism in humans.

在过去的40年,我国化学工业对国民经济的支撑比重不断增大,有毒有害物质的生产使用呈持续增高态势,其引发的环境安全问题引起公共社会的普遍担忧。其中,双酚类化合物(BPs)和对羟基苯甲酸酯类化合物(PBs)是两类高产量化学品,具有潜在的内分泌干扰效应,是目前国内外广泛关注的新污染物。BPs是一类化学结构中包含两个对位酚羟基官能团的化合物,其中使用历史最长、使用量最大的化合物是双酚A(bisphenol A, BPA)^[1]^。BPA广泛用于塑料、食品包装和其他产品。由于其显著的内分泌干扰效应^[2,3]^,世界各国已严格限制BPA的生产与使用,进而导致许多结构与功能类似的BPA替代品得到迅速开发利用,主要包括双酚B(bisphenol B, BPB)、双酚AF (bisphenol AF, BPAF)、双酚AP (bisphenol AP, BPAP)、双酚C (bisphenol C, BPC)、双酚E (bisphenol E, BPE)、双酚F (bisphenol F, BPF)、双酚P (bisphenol P, BPP)、双酚S (bisphenol S, BPS)和双酚Z (bisphenol Z, BPZ)。已有研究发现,BPA替代品同样具有一系列毒性效应,如BPB、BPAF和BPC等具有与BPA相当或更强的雌激素效能^[3,4]^, BPS和BPF具有生殖毒性^[5]^;相较于BPA及其替代品的环境污染调查研究^[6,7]^,其人体暴露负荷的报道相对较少。PBs是一系列由对羟基苯甲酸构成的酯类。PBs具有抗微生物和抗真菌活性,因其成本低、耐高温等特性,广泛应用于个人护理产品、药品、食品和饮料包装等^[8,9]^。目前商售的PBs主要有对羟基苯甲酸甲酯(methylparaben, MeP)、对羟基苯甲酸乙酯(ethylparaben, EtP)、对羟基苯甲酸丙酯(propylparaben, PrP)、对羟基苯甲酸丁酯(*n*-butyl paraben, BuP)和对羟基苯甲酸苄酯(benzylparaben, BeP)等^[9]^。尽管国际上对于PBs毒性的强弱及危害存在争议,但越来越多研究证实,PBs与多种疾病的发生相关,比如PBs暴露可提高女性乳腺癌发病率和促进恶性黑色素瘤的发展^[10,11]^。因此,国内外科学家广泛关注PBs的环境暴露特征及其对人体的潜在健康风险。

BPs和PBs主要通过膳食、呼吸和皮肤暴露进入人体^[3,9,12,13]^,由于暴露途径多、变化大,各种暴露途径对人体内暴露的贡献难以准确评估,因此,人体生物监测选取尿液中的BPs和PBs作为暴露生物标志物,用于评价两类化合物的内暴露水平^[8,13⇓⇓-16]^。由于尿液中BPs和PBs浓度处于痕量水平,且尿液基质复杂,需要通过开发准确、高灵敏度的定量分析方法来评估内暴露水平,其中富集效率高、去除基质干扰效果好的前处理技术是关键。目前常用的前处理方法有液液萃取法(liquid-liquid extraction, LLE)^[17]^、固相萃取法(solid-phase extraction, SPE)^[18]^、分散液液微萃取法(dispersive liquid-liquid microextraction, DLLME)^[19]^等,其中SPE操作过程需要经过活化固定相、加样、干扰物洗脱、待测组分收集4个步骤,耗时长,对于理化性质差异较大的不同污染物同时富集净化较难;LLE和DLLME前处理操作简单,但这两种方法容易产生乳化现象,目标物选择性差、萃取效率低,且生物基质干扰较严重^[20]^。本研究拟采用固相支撑液液萃取法(solid supported liquid-liquid extraction, SLE)对尿样进行前处理。SLE是近几年新开发的前处理方法,SLE采用特殊工艺处理的硅藻土作为固定相,该硅藻土填料具有极大的比表面积和极低的表面活性,和多种与水不相溶的有机溶剂完全兼容,能提供理想的液液分配界面。SLE操作简便,仅用上样和洗脱两步就可从水相中萃取目标物。与传统的LLE和SPE技术相比,该技术具有基质效应较低、不易产生乳化现象等优点^[21]^。SLE技术已开始应用于人体尿样和血液中目标化合物的萃取与净化,如苯二氮平类药物、醛固酮、合成麝香、羟基多环芳烃等^[22⇓-24]^,与其他方法相比,SLE具有基质干扰弱、萃取效率高、前处理时间短等优点,更适用于生物体液中痕量污染物的高通量分析。利用SLE技术开发多类污染物的同时富集净化方法,将为综合评价人体的污染物暴露水平提供有力的技术支持。

本研究选取环境中常见的10种BPs和5种PBs作为目标化合物,利用SLE柱对尿液中的15种目标物进行富集净化,筛选适合的洗脱溶剂和用量,并在此基础上,优化高效液相色谱(HPLC)的流动相和三重四极杆质谱(MS/MS)的参数,最终构建的方法用于人体尿液中目标化合物的定量分析。目前尚未见SLE柱用于人体尿样中PBs与BPs富集净化的研究报道。

## 1 实验部分

### 1.1 仪器、试剂与材料

1100型高效液相色谱仪(美国Agilent公司)-API4000三重四极杆质谱仪(美国AB SCIEX公司); Visiprep^TM^ 12孔固相萃取装置(美国Supelco公司); Heraeus^TM^ Labofuge^TM^ 200台式离心机(美国Thermo Fisher公司);氮吹仪(美国Pierce公司); 0.2 μm聚四氟乙烯膜滤头(上海安谱科学仪器有限公司); 5 mL ISOLUTE^®^ SLE^+^固相支撑液液萃取小柱(瑞典Biotage公司);Milli-Q超纯水机(美国Millipore公司)。

甲醇(MeOH)和乙腈(ACN)购自德国Merck公司,正己烷(Hex)、乙酸乙酯(EtAc)、二氯甲烷(DCM)和甲基叔丁基醚(MTBE)购自德国CNW Technologies公司,以上试剂均为色谱纯;无水乙酸钠(NaAc,纯度99.0%)和氨水(NH_3_·H_2_O, 28%~30%)购自上海安谱实验科技股份有限公司;乙酸(HAc, 99.97%)购自美国Tedia公司;*β*-葡萄糖苷酸-芳基硫酸酯混合酶(每毫升含122400单位*β*-葡糖苷酸酯酶和3610单位芳基硫酸酯酶)购自美国Aldrich-Sigma化学试剂公司;10种BPs单标(BPA、BPAF、BPAP、BPB、BPC、BPE、BPF、BPP、BPS与BPZ,纯度>98%)和5种PBs单标(MeP、EtP、PrP、BuP与BeP,纯度>98%)均购自美国AccuStandard公司。4种回收率指示物^13^C_12_-BPS(纯度98%)、^13^C_12_ -BPAF(纯度99%)、^13^C_6_-MeP(纯度99%)与^13^C_6_-BuP(纯度99%)购自美国Cambridge Isotope Laboratories。15种目标化合物名称和结构见图1。

**图1:**
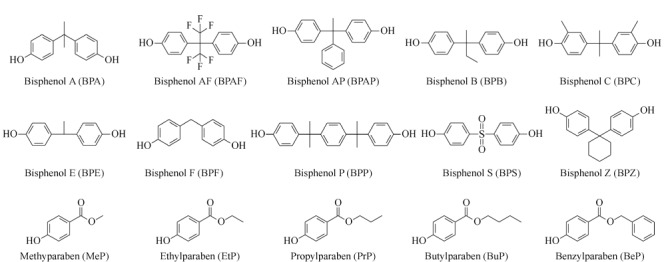
10种双酚类和5种对羟基苯甲酸酯的化学结构

### 1.2 尿液样品采集

方法优化用尿样:随机取实验室志愿者尿液10例,每例取10 mL进行混合,形成混合尿液基质,采样时间为2023年4月。方法验证用尿样:随机选取10例人群尿液样本进行检测,该尿液样品的采集对象为广东省普通人群,采样时间为2014-2015年,所有采集对象均自愿参加并签署同意书。尿液样本采集后保存于预先处理过的聚乙烯塑料瓶中,放置于-80 ℃冰箱。本研究通过了南方医科大学伦理审查委员会论证审查(批件号:NFEC-2015-106)。

### 1.3 溶液配制

分别准确量取上述15种目标化合物和4种同位素标记物单标,使用甲醇配制质量浓度均为1000 μg/L的混合标准储备液。同样的方法单独配制4种回收率指示物混合标样,用甲醇稀释至500 μg/L备用。使用逐级稀释法,将适量混合标准储备液用甲醇稀释,配制质量浓度范围为0.1~500 μg/L的标准溶液;每个浓度点准确量取200 μL标准溶液转移到按1.4节中方法处理好的干燥空白尿样基质中,制作基质匹配的标准曲线用于定量分析;配制质控样,包含10种BPs(100 μg/L)、5种PBs(50 μg/L)和4种回收率指示物(5 μg/L)。使用之前,所有标准品(包括标准品储备液、标准溶液和质控样等)置于-20 ℃条件下保存。

### 1.4 尿样酶解与目标物萃取

将样品于室温下解冻后,以3000 r/min离心10 min后取上清液,准确量取2 mL并分别加入20 μL质量浓度为500 μg/L的回收率指示物混合标样后,加入0.5 mL 0.1 mol/L HCl溶液调节尿液pH至5.0,随后依次加入1.5 mL NaAc-HAc缓冲溶液、20 μL *β*-葡萄糖苷酸-芳基硫酸酯酶,涡旋充分混匀后置于恒温振荡器中于37 ℃下避光酶解16 h。

将酶解后的尿液样品加入固相支撑液液萃取柱中,等待5 min使样品分散、充分吸附于填料表面。连续3次用5 mL EtAc-Hex(3∶7, v/v)混合溶液进行洗脱,洗脱液合并收集后通过高纯氮气缓慢吹干,最后用甲醇定容至200 μL后放入-20 ℃冰箱保存,待检测分析。

### 1.5 液相色谱-质谱条件

色谱柱为Zorbax SB-C18柱(250 mm×4.6 mm, 5 μm, Agilent, USA),甲醇、乙腈和水为流动相,液相色谱流动相梯度淋洗程序见表1。进样量:20 μL;柱温:30 ℃;流动相初始流速400 μL/min;离子源:电喷雾电离源(ESI);电离方式:负电离;扫描方式:多反应监测(MRM)模式;离子源温度:450 ℃;喷雾电压:-4500 V;辅助气压力:414 kPa;雾化气压力:344 kPa;帘气压力:207 kPa。15种目标物及4种同位素标记的回收率指示物的最优监测离子对、去簇电压和碰撞能等质谱参数见表2。

**表1:** 15种目标分析物的流动相梯度洗脱程序

Time/ min	Flow rate/ (μL/min)	Volume fractions/%		
		Water	Methanol	Acetonitrile
0	400	35	55	10
5	400	35	55	10
10	400	30	60	10
20	600	10	70	20
22	600	0	70	30
28	600	0	70	30
31	400	35	55	10
40	400	35	55	10

**表2:** 15种目标物及4种回收率指示物的保留时间和质谱参数

Analyte	Retention time/min	Ion pairs (*m/z*)	Declustering potentials/V	Collision energies/eV
BPS	7.7	249.1>108.0^*^, 249.1>156.0	-60, -60	-30, -29
BPF	11.1	198.9>92.90^*^, 198.9>105.0	-73, -69	-35, -24
BPE	13.1	213.0>198.0^*^, 213.0>197.0	-45, -80	-26, -40
BPA	15.4	226.9>212.0^*^, 226.9>132.9	-72, -71	-32, -22
BPB	18.6	241.0>211.1^*^, 241.0>225.9	-74, -83	-29, -29
BPAF	19.3	335.0>265.1^*^, 335.0>315.0	-57, -60	-30, -27
BPAP	19.9	289.1>273.8^*^, 289.1>194.6	-77, -79	-30, -27
BPC	20.4	255.3>147.0^*^, 255.3>240.0	-52, -58	-35, -35
BPZ	21.9	267.2>173.2^*^, 267.2>223.1	-60, -59	-39, -38
BPP	25.7	344.9>330.3^*^, 344.9>132.7	-65, -63	-35, -34
^13^C_12_-BPS	7.2	262.9>113.9^*^, 262.9>164.1	-65, -60	-40, -40
^13^C_12_-BPAF	19.3	346.1>276.1^*^, 346.1>69.2	-60, -60	-50, -50
MeP	8.6	151.1>91.8^*^, 151.1>135.9	-55, -55	-40, -40
EtP	10.4	165.1>91.8^*^, 165.1>135.9	-62, -59	-36, -36
PrP	14.0	179.1>91.8^*^, 179.1>135.9	-56, -50	-28, -24
BuP	19.6	193.1>91.8^*^, 193.1>135.9	-62, -52	-32, -30
BeP	18.8	227.1>91.8^*^, 227.1>135.9	-60, -62	-40, -34
^13^C_6_-MeP	8.6	155.9>97.0^*^, 155.9>140.9	-64, -60	-42, -32
^13^C_6_-BuP	19.5	198.8>97.4^*^, 198.8>142.8	-57, -50	-34, -30

* Quantitative ion pair.

### 1.6 质量控制与质量保证

为避免实验本底干扰,实验室用的玻璃器皿均使用酸性重铬酸钾清洗,于450 ℃马弗炉中烘烤,使用前所有器皿用超纯水和甲醇各润洗两遍。每6个样品一组进行预处理,每组样品中添加一个程序空白样品,用来监控实验室处理过程中潜在的背景污染。4种回收率指示物用于指示尿样中目标物的萃取效果。程序空白用2 mL超纯水作为基质,其他处理过程与尿样一致。仪器分析时,每10个样品之间插入一个甲醇空白样以监测仪器背景。

## 2 结果与讨论

### 2.1 液相色谱-质谱条件的优化

#### 2.1.1 质谱参数优化

本研究中BPs与PBs的质谱参数优化依据如下原则:首先在全扫描(full scan)模式下,设定扫描质荷比(*m/z*)范围为50~400,确定每个BPs与PBs的母离子峰(一般为准分子离子峰),随后进行子离子峰扫描(MS^2^ scan),确定每个化合物的定性和定量离子对;在此基础上采用MRM,对各化合物定性与定量离子对的相关参数进行优化,最终优化的结果见表2。

#### 2.1.2 色谱条件的优化

由于本研究中15种目标化合物在人体尿液中的浓度差异大,为兼顾低浓度化合物的分析灵敏度,增大进样体积是简便有效的办法,故实验选用柱容量较大的Zorbax SB-C18反相色谱柱(250 mm×4.6 mm, 5 μm)作为目标化合物的分析柱,并通过流动相的梯度洗脱和流速的变化获得最佳色谱条件。MeOH和ACN是常用的有机流动相,广泛用于反相液相色谱中弱极性化合物的色谱分离。相较而言,MeOH作为质子性溶剂,可提高化合物的质谱响应,而ACN在相同流速下具有更低的柱背压以及更强的洗脱能力,可带来更高的柱效^[25]^。10种BPs、5种PBs的理化性质相差较大,在色谱柱上的保留时间差异大,分析时间长。实验通过不断优化条件发现,在10~20 min,流动相流速从400 μL/min缓慢升高到600 μL/min,在10~22 min,流动相中ACN的组成从10%缓慢提升到30%,可实现15种目标物的高效分析(表1)。由于ACN的洗脱能力较MeOH更强、背压更低,因此随着ACN在三元体系中含量的不断增加,一方面弱极性的化合物可缩短在液相色谱柱上的相对保留时间;另一方面ACN的引入可减少色谱柱压力变化,降低色谱柱回到初始流动相的平衡时间,从而在保证分析方法稳定性的同时缩短分析时间。

本研究还进一步考察了在流动相水相中添加常用缓冲剂的分析效果。仪器分析结果显示,在水相中添加0.5‰甲酸时,5种PBs响应有一定提高,但是添加0.5‰甲酸会显著抑制除BPS与BPAF之外其他BPs的离子化效率,化合物的灵敏度均显著下降;选用不同浓度甲酸铵-氨水作为流动相水相的缓冲盐时,会导致BPS出现双峰,且响应值降低,因此最终选用纯水为流动相水相对15种化合物进行分析。混合标准品中各化合物的色谱图见图2。

**图2:**
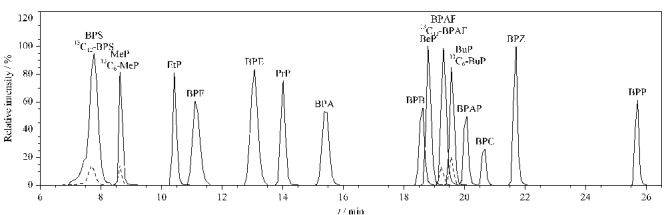
混合标准溶液中10种BPs(100 μg/L)、5种PBs(50 μg/L)和4种回收率指示物(5 μg/L,虚线)的色谱图

### 2.2 基于SLE的前处理条件优化

所购买的SLE柱采用了特殊工艺处理过的硅藻土,用于该净化柱的洗脱溶剂需要与水不混溶。溶剂的选择取决于目标物的溶解度和极性等理化性质,对于非极性化合物,可选择庚烷、戊烷或Hex等溶剂,对于弱极性化合物,则通常采用MTBE、DCM或EtAc等。本研究中,BPs的p*K*_a_值为7.64~10.31, PBs的p*K*_a_值为8.17~8.59,具有弱极性,除BPS以外,其他目标化合物的水溶解度均相对较低,故选取Hex、DCM、EtAc与MTBE 4种溶剂,每种溶剂设置平行实验3次,进行洗脱溶剂的优化,并通过15种化合物的回收率进行评价。由于用于方法优化的混合尿样中存在一定量的本底值,前处理回收率(recovery, Rec)和基质效应(matrix effect, ME)分别经公式(1)和公式(2)评估:


(1) $$\mathrm{Rec}=\frac{A_{1}-A_{0}}{A_{2}-A_{0}}\times 100\%$$

其中,*A*_0_代表前处理后非加标混合尿样中化合物的峰面积;*A*_1_代表前处理前加标的混合尿样中化合物的峰面积;*A*_2_代表前处理后加标的混合尿样中化合物的峰面积。


(2) $$ME=\frac{A_{2}-A_{0}-A_{3}}{A_{3}}\times 100\%$$

其中,*A*_3_代表纯溶剂中同浓度标准品的峰面积。ME>0,表示有基质增强效应;ME<0,表示有基质抑制效应;ME=0,表示无基质效应。

图3a给出了4种洗脱溶剂(20 mL)对回收率的影响,从图3a中可以看出,4种溶剂对于BPs与PBs的萃取效果差异较大,其中非极性的有机溶剂Hex对各目标化合物的洗脱效率均最低,回收率为0.1%~17.9%; DCM、MTBE与EtAc的极性逐步增强,其回收率也相对较高,其中EtAc对各目标物的提取效果均较理想,回收率范围为81.5%~121.1%。但随着洗脱溶剂极性的提高,洗脱出的尿液基质也会相应增多,造成较强的基质效应。由图3b可以看出,当采用极性溶剂EtAc和MTBE作为洗脱溶剂时,各目标化合物呈现较强的基质抑制效应,而采用Hex和DCM为洗脱溶剂时,基质抑制效应较弱,部分目标化合物呈现一定的基质增强效应。

**图3:**
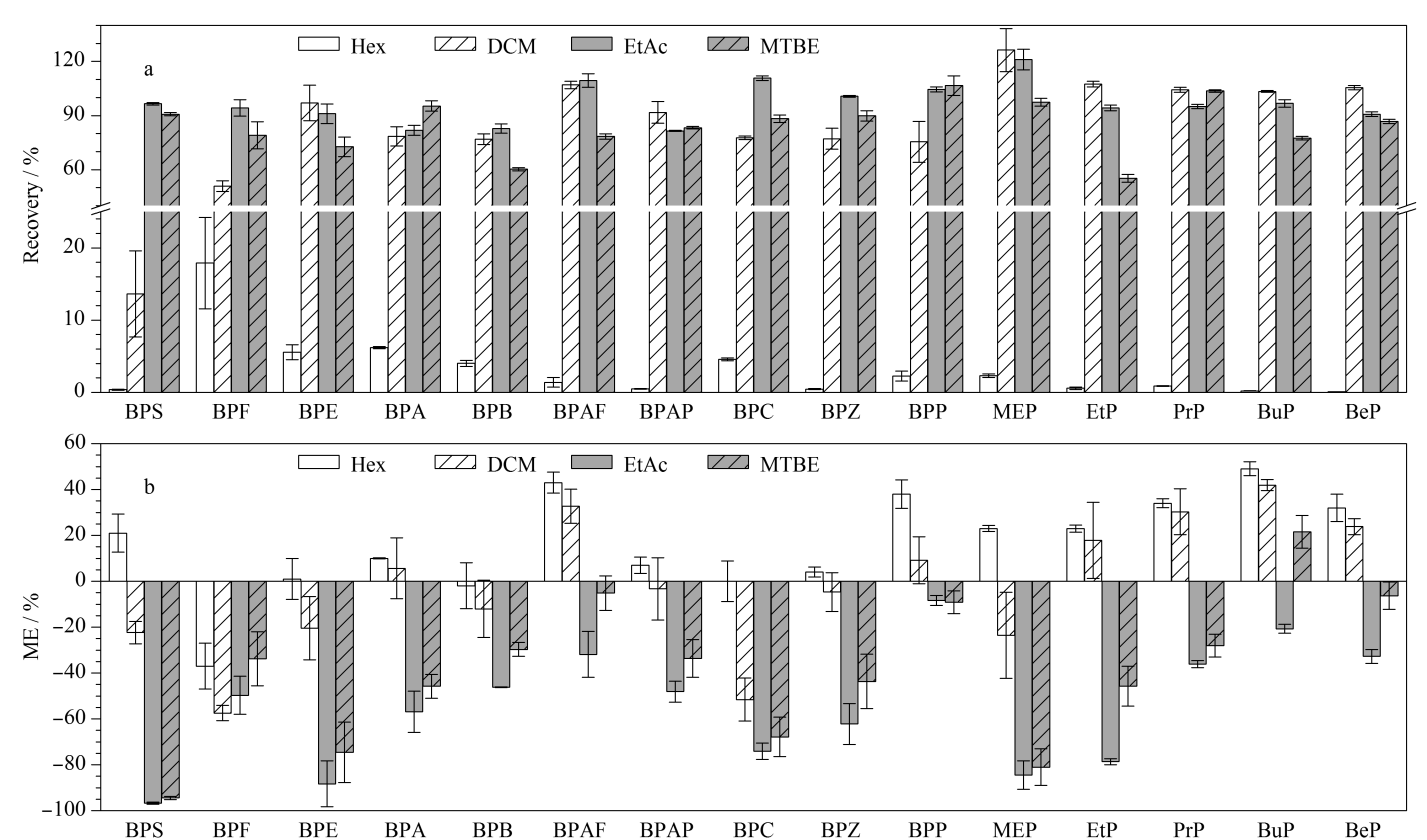
不同洗脱溶剂对目标化合物的(a)回收率以及(b)基质效应(*n*=3)

考虑到使用非极性的有机溶剂Hex时色谱峰干扰少,且Hex沸点低、浓缩时易挥发,可减少目标化合物的损失,故本研究进一步考察了EtAc-Hex不同配比时的洗脱能力和基质效应(图4)。图4a显示,当洗脱溶剂EtAc-Hex的配比为3∶7(v/v)时,所有目标物的回收率较理想,回收率范围为84.1%~121.5%。当洗脱溶剂中EtAc的含量增加时,整体回收率无明显提高,而部分化合物(如BPF、BPE、BPP等)回收率下降;当洗脱溶剂中EtAc的含量下降时,大部分化合物回收率明显降低,尤其是极性较高的BPS与BPF,其回收率均低于60%。图4b显示了不同配比洗脱溶剂对应的基质效应,随着洗脱溶剂中EtAc的含量降低,基质抑制效应随之减弱。综合考虑混合洗脱溶剂EtAc-Hex不同配比时的回收率和基质干扰情况,最终选择EtAc∶Hex=3∶7(v/v)混合溶剂作为洗脱溶剂,此时的基质效应范围为-23.0%~9.4%。

**图4:**
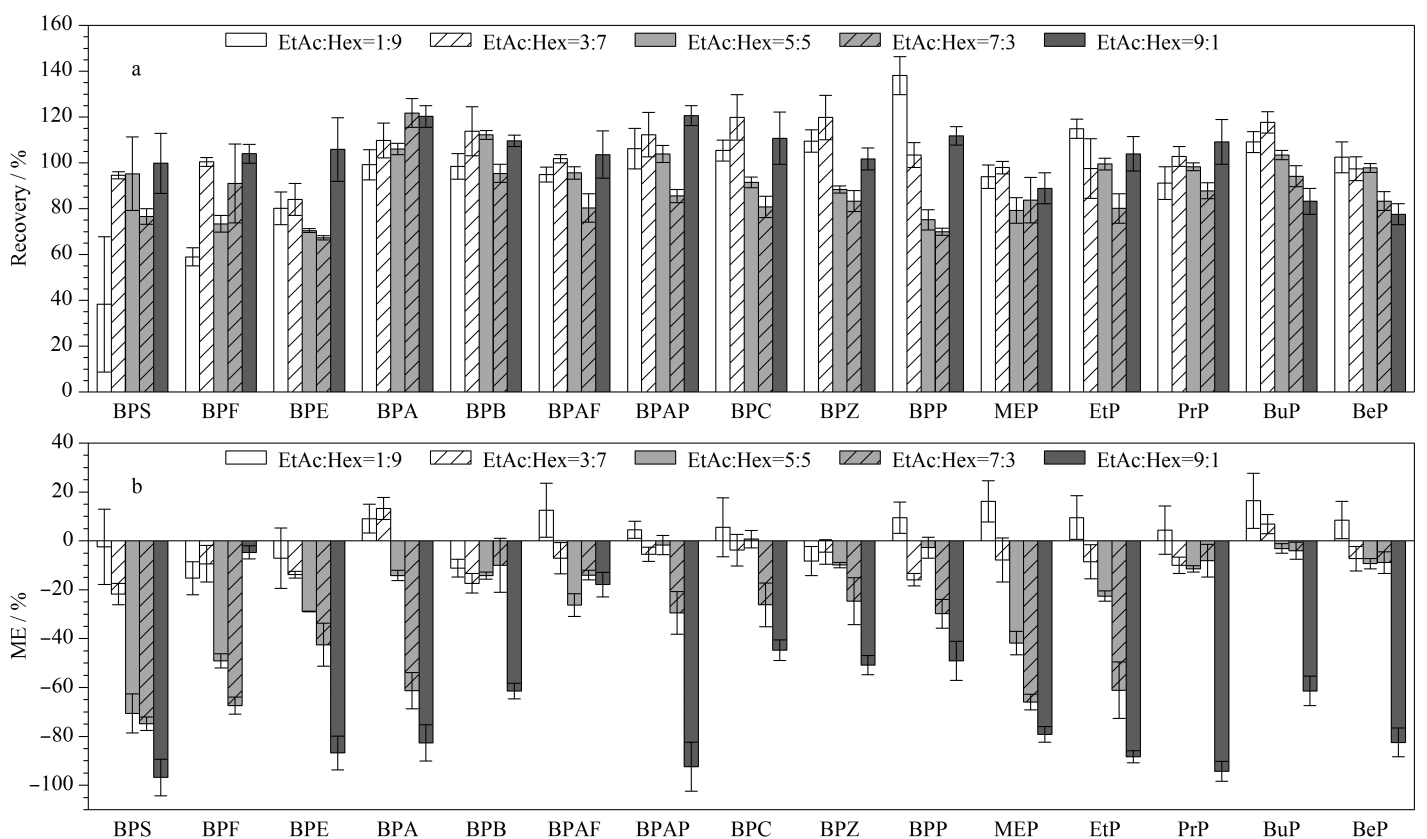
混合洗脱溶剂EtAc与Hex不同配比对目标化合物(a)回收率以及(b)基质效应的影响(*n*=3)

同时,本研究对洗脱溶剂的使用量进行了优化。实验中每5 mL收集一次洗脱液,得到每个5 mL洗脱的目标物回收率占比(图5)。x从图5可以看出,随着洗脱溶剂用量的增加,其每5 mL洗脱下来的目标化合物含量依次降低,第四个5 mL时目标化合物已低于仪器检出限,因此最终选择洗脱溶剂用量为15 mL。

**图5:**
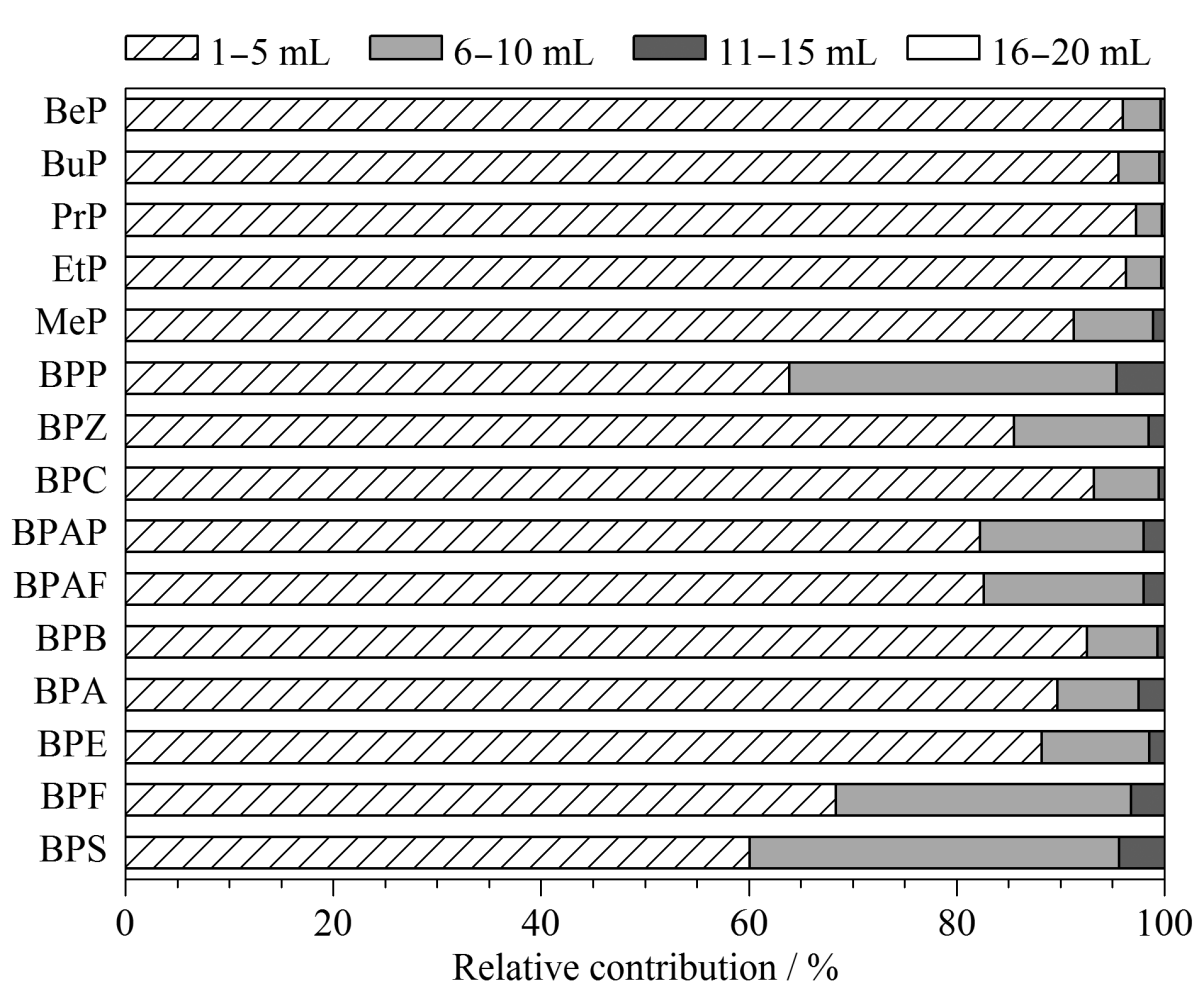
洗脱溶剂体积对目标回收率的影响

### 2.3 方法评价

文献资料显示,我国普通人群尿液中的PBs和BPs浓度较低,检出率较高的BPA、BPS和MeP的中位数含量范围分别是0.84~5.08 μg/L、0.04~0.299 μg/L和3.8~23.1 μg/L,其他化合物含量中值大多处于定量限水平^[3,26⇓⇓⇓-30]^,故方法评价中设定低(1 μg/L)、中(5 μg/L)和高(50 μg/L)3个水平进行加标试验,每组实验重复6次。从基质效应、线性范围、回收率、重复性、精密度、方法检出限(MLD)和方法定量限(MLQ)等几个方面对建立的方法进行评价。

在最优前处理条件和测定条件下对方法开展基质效应的评价,结果表明,除了BPA显示基质增强效应,其余化合物均呈基质抑制效应,基质效应为-21.8%~13.3%,其中BPS的基质抑制效应超过了20%,这可能是由于BPS的极性和水溶性较大,在液相色谱分离时,保留时间靠前而受非保留基质的影响较大造成的。

由于基质效应会对分析结果的准确性产生影响,本实验选取5个不同来源的尿样(目标物本底值低于检出限)按照前处理方法处理获得的洗脱液为基质,配制基质匹配的标准曲线进行定量分析,以目标化合物的质量浓度为横坐标,目标化合物色谱峰面积为纵坐标,绘制标准曲线。如表3所示,15种目标化合物线性相关系数(*R*^2^)均在0.995以上,表明线性关系良好。

**表3:** 10种BPs和5种PBs的线性关系、方法检出限、方法定量限及精密度

Compound	Linear range/ (μg/L)	Regression equation	Correlation coefficient (*R*^2^)	MLD/ (μg/L)	MLQ/ (μg/L)	Precisions/% (*n*=6)	
						Intra-day	Inter-day
BPS	0.1-500	*y*=541515*x*+35600	0.9990	0.01	0.04	2.9	5.9
BPAF	0.1-500	*y*=942480*x*+12202	0.9955	0.01	0.03	4.6	8.2
BPF	1-500	*y*=17450*x*-3820	0.9993	0.09	0.30	6.2	11.6
BPAP	0.5-500	*y*=112907*x*-4067	0.9990	0.03	0.08	3.7	9.3
BPZ	0.5-500	*y*=60166*x*-5078	0.9992	0.05	0.12	8.4	14.6
BPA	1-500	*y*=24993*x*-7973	0.9986	0.06	0.20	6.1	12.9
BPB	1-500	*y*=18558*x*-4143	0.9992	0.05	0.18	4.3	8.0
BPE	1-500	*y*=65503*x*-15277	0.9994	0.09	0.30	3.9	10.1
BPC	1-500	*y*=14458*x*-2714	0.9993	0.10	0.30	6.7	12.9
BPP	0.5-500	*y*=53449*x*+8589	0.9976	0.10	0.20	3.5	9.4
MeP	0.1-500	*y*=348826*x*+61668	0.9989	0.01	0.03	2.9	6.5
EtP	0.1-500	*y*=380128*x*+63576	0.9990	0.01	0.04	2.3	5.7
PrP	0.1-500	*y*=413702*x*+40933	0.9997	0.01	0.03	1.9	9.7
BuP	0.1-500	*y*=448783*x*+62501	0.9995	0.01	0.03	3.0	10.3
BeP	0.1-500	*y*=503662*x*+12123	0.9998	0.01	0.03	1.4	8.6

*y*: peak area of the quantitative ion of the analytes; *x*: mass concentration, μg/L.

精密度结果显示,BPs(100 μg/L)与PBs(50 μg/L)的日内精密度(*n*=6)范围为1.4%~8.4%,日间精密度(连续6天)范围为5.7%~14.6%,表明分析方法稳定可靠。

MLD和MLQ的计算使用基质匹配标准溶液,浓度从高到低不断稀释进行仪器分析,设定色谱图上目标峰3倍和10倍信噪比对应的浓度分别为仪器检出限和仪器定量限,并根据方法中尿液用量和定容体积换算为方法检出限和方法定量限。本实验中15种目标物的方法检出限为0.01~0.10 μg/L,方法定量限为0.03~0.30 μg/L。本方法的回收率评价采用标准加入法进行考察,每个加标浓度进行6次重复实验,加标回收率的计算方法如下:根据基质匹配的标准曲线计算成质量浓度后,用加标混合尿液的浓度值与未加标混合尿液的背景浓度值之差除以实际加标浓度,再转换为百分数。表4给出了BPs和PBs的回收率。实验结果显示,各目标化合物在3个加标浓度下的回收率为84.3%~119.8%,标准偏差为2.7%~16.9%,RSD为2.3%~19.1%,表明该方法具有良好的加标回收率和稳定性。

**表4:** 3个加标水平下BPs与PBs的回收率及标准偏差(*n*=6)

Compound	1 μg/L	5 μg/L	50 μg/L
BPS	90.5±10.7	94.6±4.6	93.3±7.5
BPF	101.3±12.5	99.3±4.8	105.8±12.4
BPE	103.1±13.2	84.3±7.4	88.9±7.9
BPA	106.2±16.3	101.3±12.1	101.2±13.8
BPB	96.6±9.3	105.1±13.6	101.1±7.3
BPAF	111.2±8.9	101.2±3.9	91.9±4.1
BPAP	104.3±9.2	104.8±11.1	102.5±7.9
BPC	101.1±5.4	101.5±13.2	87.1±10.1
BPZ	104.7±11.7	108.8±13.9	103.2±4.9
BPP	88.5±16.9	101.0±7.1	89.4±11.2
MeP	107.9±5.8	98.2±2.3	93.8±4.9
EtP	101.4±10.8	100.5±11.3	88.4±7.9
PrP	101.5±5.9	96.7±8.4	85.9±5.2
BuP	119.8±13.2	107.9±12.7	92.6±2.7
BeP	108.7±6.9	96.5±4.8	99.6±3.9

### 2.4 方法应用

采用本方法对10例尿液样本进行BPs和PBs的检测,4种同位素标记的回收率指示物^13^C_12_-BPS、^13^C_12_-BPAF、^13^C_6_-MeP和^13^C_6_-BuP的回收率分别为(85.1±10.1)%、(92.9±9.1)%、(93.4±7.1)%和(96.3±9.6)%,表明分析结果可靠。表5显示,MeP、EtP、PrP和BPA在人体尿样中普遍检出,其余化合物检出率低于50%,不同化合物在人体内的暴露水平呈现显著差异,这可能与化合物的环境浓度以及生产使用量相关,也与化合物的生物可利用性以及在人体内的生物代谢能力相关。MeP、EtP、PrP和BPA的质量浓度中值分别为1.10、0.60、0.21和0.55 μg/L。研究结果证实,本方法可用于人体尿液中BPs和PBs的检测。随着未来研究中样本库的扩大,本方法可发挥其前处理时间短、回收率高、基质干扰相对较低的特点,用于环境流行病学的大队列人群研究。

**表5:** 10例普通人群尿样中PBs和BPs的含量

Sample No.	5 PBs						10 BPs									
	MeP	EtP	PrP	BuP	BeP		BPS	BPAF	BPP	BPC	BPE	BPB	BPF	BPAP	BPZ	BPA
S1	0.74	0.12	0.03	<MLQ	<MLQ		0.05	0.05	<MLQ	0.65	0.35	-	-	-	-	0.24
S2	1.15	1.68	0.35	<MLQ	-		0.13	<MLQ	<MLQ	-	-	-	<MLQ	<MLQ	-	0.54
S3	1.12	1.46	0.15	<MLQ	0.05		-	0.04	<MLQ	<MLQ	<MLQ	-	-	0.08	<MLQ	0.25
S4	0.53	0.20	0.05	<MLQ	-		-	<MLQ	-	<MLQ	<MLQ	-	<MLQ	<MLQ	<MLQ	0.32
S5	6.94	0.12	12.21	1.12	-		-	<MLQ	<MLQ	<MLQ	-	-	<MLQ	-	-	0.44
S6	0.66	0.15	0.03	<MLQ	-		0.04	0.05	<MLQ	<MLQ	-	-	<MLQ	0.08	-	1.12
S7	1.08	0.55	0.19	<MLQ	0.05		-	<MLQ	<MLQ	0.58	<MLQ	-	<MLQ	0.09	-	1.44
S8	0.66	0.26	0.03	0.04	-		-	<MLQ	<MLQ	<MLQ	-	-	<MLQ	-	<MLQ	0.48
S9	9.23	14.13	0.23	<MLQ	-		0.05	0.06	-	<MLQ	-	-	<MLQ	<MLQ	-	0.56
S10	6.56	0.66	0.25	<MLQ	-		-	<MLQ	<MLQ	<MLQ	<MLQ	-	6.54	<MLQ	-	0.33

-: not detected.

## 3 结论

基于构建多种污染物同时前处理技术、满足高通量样品分析的研究需求,本文利用硅藻土SLE柱,建立了同时富集、净化人体尿液中10种BPs和5种PBs的前处理方法。通过筛选优化,最终确定采用乙酸乙酯-正己烷(3∶7, v/v)的混合溶剂进行洗脱。同时通过优化三元流动相和质谱参数,最终实现10种BPs和5种PBs的准确定性和定量分析。新建立的方法具有操作简单快速、回收率高、生物基质干扰弱等特点,可满足人体尿液中BPs、PBs的同时检测。
